# Recent Advances in Piezoelectric Wafer Active Sensors for Structural Health Monitoring Applications

**DOI:** 10.3390/s19020383

**Published:** 2019-01-18

**Authors:** Hanfei Mei, Mohammad Faisal Haider, Roshan Joseph, Asaad Migot, Victor Giurgiutiu

**Affiliations:** 1 Department of Mechanical Engineering, University of South Carolina, 300 Main Street, Columbia, SC 29208, USA; haiderm@email.sc.edu (M.F.H.); rjoseph@email.sc.edu (R.J.); amigot@email.sc.edu (A.M.); victorg@mailbox.sc.edu (V.G.); 2 Department of Mechanical Engineering, College of Engineering, Thi-Qar University, Nasiriyah 64001, Iraq

**Keywords:** structural health monitoring, piezoelectric wafer active sensors, active sensing, passive sensing, damage detection, acoustic emission

## Abstract

In this paper, some recent piezoelectric wafer active sensors (PWAS) progress achieved in our laboratory for active materials and smart structures (LAMSS) at the University of South Carolina: http: //www.me.sc.edu/research/lamss/ group is presented. First, the characterization of the PWAS materials shows that no significant change in the microstructure after exposure to high temperature and nuclear radiation, and the PWAS transducer can be used in harsh environments for structural health monitoring (SHM) applications. Next, PWAS active sensing of various damage types in aluminum and composite structures are explored. PWAS transducers can successfully detect the simulated crack and corrosion damage in aluminum plates through the wavefield analysis, and the simulated delamination damage in composite plates through the damage imaging method. Finally, the novel use of PWAS transducers as acoustic emission (AE) sensors for in situ AE detection during fatigue crack growth is presented. The time of arrival of AE signals at multiple PWAS transducers confirms that the AE signals are originating from the crack, and that the amplitude decay due to geometric spreading is observed.

## 1. Introduction

Structural health monitoring (SHM) is an emerging interdisciplinary research field, which aims at detecting damage and providing a diagnosis of structural health [1,2,3,4,5]. Among the SHM technologies, Lamb wave, a type of ultrasonic guided waves propagating between two parallel surfaces without much energy loss, is suitable for the large-area inspection of complicated structures [6,7]. Piezoelectric wafer active sensors (PWAS) were developed by our LAMSS group as convenient enablers for generating and receiving Lamb waves in structures for SHM applications [8]. 

Depending on the type of application, PWAS can be utilized for (i) active sensing of far-field damage using pulse-echo, pitch-catch, and phased-array methods, (ii) active sensing of near-field damage using the electromechanical impedance method, and (iii) passive sensing of acoustic emissions at the tip of advancing cracks and low-velocity impacts [8]. The main advantage of PWAS transducers over conventional ultrasonic probes is their low cost and light weight. They can be permanently bonded on the host structures in large quantities, and achieve real-time monitoring of the structural health status. These PWAS transducers can also be used in a harsh environment (e.g., high temperature, nuclear radiation, or a vacuum environment). A proper sensor characterization would be required before its installation on the host structure in a harsh environment. 

In recent years, many researchers have explored the capability of PWAS for SHM applications, such as the characterization of PWAS [9,10,11,12,13], impact localization [14,15,16,17,18,19], acoustic emission (AE) detection [20,21], and damage detection in isotropic and composite plates [22,23,24,25,26,27]. These studies facilitate the understanding of PWAS-based SHM applications. 

In order to use the PWAS transducer as an SHM transducer in harsh environments, PWAS material properties should be investigated, and PWAS transducers should be defect-free after high temperature and radiation exposure. Baptista et al. [9] conducted an experimental study of the effect of temperature on the electrical impedance of the piezoelectric sensors, and they found that the temperature effects were strongly frequency-dependent. Similarly, Haider et al. [10] investigated the irreversible change in PWAS electromechanical (E/M) impedance and admittance signature under high-temperature exposure. It was concluded that the changes in anti-resonance and resonance frequencies have a linear relationship with temperature. In addition, the sensitivity characterization of high temperature piezoelectric transducers was performed by using resonance analysis [11]. Sinclair and Chertov [12] presented a comprehensive literature study of the radiation endurance of a piezoelectric transducer. 

General PWAS sensing technology can be cast into two methodological categories: passive sensing and active sensing. PWAS passive sensing methods only record events passively, which happens during the period of interest. By analyzing the recorded signal, diagnosis can be made on the health status of the structure. Examples of PWAS passive sensing methods can be found in the impact localization [15,18] and AE detection [20,21]. Park et al. [15] proposed a new technique for predicting the impact location on an anisotropic plate by analyzing the geometric shape of the wavefront, and it did not require prior knowledge of the material properties. Qiu et al. [18] conducted an impact localization on an aircraft composite wing box using piezoelectric sensor networks, and achieved promising results. Apart from impact localization using PWAS transducers, preliminary work has also been performed to capture fatigue AE hits [20,21]. 

In contrast to PWAS passive sensing, PWAS active sensing methods interrogate the structures with defined excitations and record the corresponding response. One common active sensing method in SHM applications is the pitch-catch experiment, where one PWAS transducer acts as the transmitter and sends out Lamb waves, and another PWAS transducer acts as the receiver and picks up the sensing signal. The subtraction between the pristine and damaged responses may indicate the presence and severity of the damage. To achieve more complicated diagnostic approaches for SHM applications, several sensors may work together in a systematically designed manner, forming a sensor network. Advanced damage imaging techniques have been developed by using the phased array [28,29] and sparse array [30,31,32,33,34,35,36,37,38,39,40,41,42,43,44,45,46,47,48,49]. Substantial research has focused on the research of damage imaging algorithms for damage localization and characterization, using a sparse PWAS array, including tomography [30,31,32], the delay-and-sum imaging method [33,34,35], the time-reversal imaging approach [36,37,38], correlation-based imaging algorithm [39,40], probabilistic and statistical imaging methods [41,42,43], and the minimum variance imaging method [44,45,46]. Recently, Kudela et al. [48] proposed a Lamb wave-focusing method to detect and visualize the crack in an aluminum plate, and the damage imaging resolution was improved compared to the original delay-and-sum algorithm. Xu et al. [49] developed a weighted sparse reconstruction-based anomaly imaging method for damage detection on composite plates, and anomaly imaging with fewer artifacts was achieved.

Moreover, various methods have been used for wavefield analysis. In this active sensing method, one PWAS transducer was used to excite Lamb waves propagating in the structures, and the wavefield was measured by a scanning laser Doppler vibrometer (SLDV). The damage can be unveiled through wavefield analysis, such as the wavefield amplitude profile [49], frequency-wavenumber filtering [50,51,52,53], standing wave filtering [54,55,56], zero-lag cross-correlation imaging [57,58], local wavenumber analysis [59,60,61], and wavenumber adaptive image filtering [62,63,64]. Staszewski et al. [49] used the amplitude profiles of the wavefield to detect the delamination damage in composites. Sohn et al. [54] proposed a standing wave filter to isolate the standing waves from the propagating waves for delamination detection in composites. He and Yuan [57] employed the zero-lag cross-correlation (ZLCC) imaging condition for damage imaging in a composite plate, using a single piezoelectric wafer for excitation. In recent years, various wavenumber imaging methods were applied for impact-induced delamination detection. Girolamo et al. [58] applied the ZLCC imaging condition for visualizing impact damage in a honeycomb composite panel. Rogge and Leckey [59] presented a local wavenumber domain analysis to process the wavefield, and they demonstrated that it could be used to quantify the delamination depth and size. Tian et al. [60,61] improved the damage visualization algorithm by using filtering reconstruction imaging and spatial wavenumber imaging. Kudela et al. [64] studied the relation between impact energy and BVID detectability, using wavenumber adaptive image filtering, and they found that damage caused by the impact of 10 J or higher could be successfully detected. 

In this paper, some recent PWAS progress achieved in our LAMSS group is reported, including studies of (a) PWAS endurance in harsh temperature and radiation environments; (b) PWAS active sensing of various damage types in aluminum and composite structures; (c) PWAS passive sensing of acoustic emission (AE) signals from fatigue crack growth in aluminum coupons. First, the characterization of PWAS materials is conducted. The endurance of the PWAS after exposure to high temperature and nuclear radiation was also assessed for harsh environmental applications. Next, applications of PWAS, using active sensing methods for detecting the simulated crack and corrosion damage in aluminum plates, and the simulated delamination damage in composite plates are conducted. Finally, the novel use of PWAS transducers as AE sensors for in situ AE detection during fatigue crack growth is presented. 

## 2. Characterization of PWAS 

**Figure 1:**
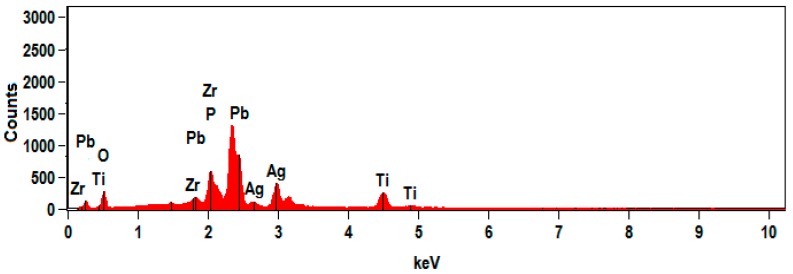
Energy-dispersive spectroscopy (EDS) spectrum of the lead zirconate titanate (PZT) piezoelectric wafer active sensors (PWAS).

In this section, PWAS transducers made with piezoelectric material lead zirconate titanate (PbZrO_3_TiO_3_ or PZT) were investigated. PZT exhibits large electromechanical coupling coefficients and piezoelectric constants [65]. For PZT-PWAS, APC 850 type transducers [66] were used in this research. The PZT-PWAS transducer is circular in shape, and the diameter is 7 mm. The wafer has a PZT thin film with Ag electrodes on both sides. The thickness of the PZT-PWAS transducer is 0.2 mm. Energy-dispersive spectroscopy (EDS) was done on the PWAS transducer to obtain chemical compositions of the PWAS. Figure 1 shows the EDS spectrum of PZT-PWAS. The figure confirms that PZT-PWAS contains lead (Pb), zirconium (Zr), titanium (Ti), oxygen (O), and silver (Ag) electrode.

**Figure 2:**
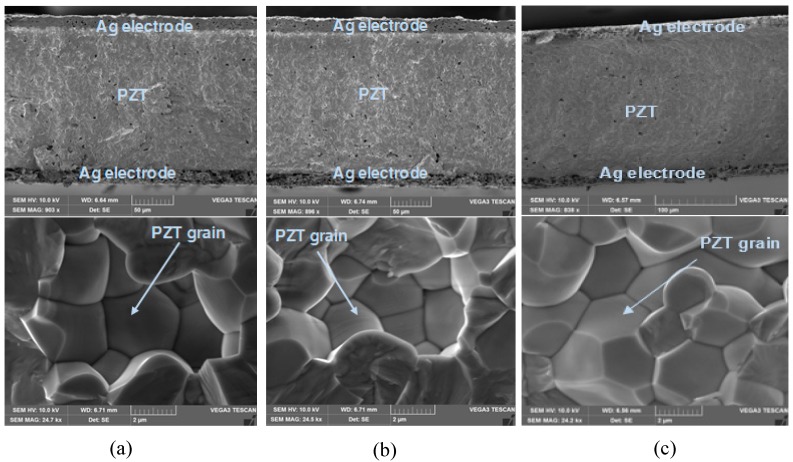
Scanning electron microscopy (SEM) image of the cross-section of PZT-PWAS: (**a**) at room temperature; (**b**) after exposure to 250 °C temperature; (**c**) after exposure to 225 kGy nuclear radiation.

The significance of SHM has been emphasized in many fields, such as dry cask storage canister (nuclear-spent fuel storage), pressure vessel and pipe (PVP), turbine blade and so on; where attention is being drawn to the successful implementation of SHM techniques, due to temperature variation and radiation. The PZT material used in a PWAS transducer is a ferroelectric material. For most ferroelectric materials, the existence of a domain structure or domain wall makes a significant influence on the material properties. In a PZT solid-solution system, the material properties may be changed, due to the change in the domain size and the domain wall motion [67,68,69]. In addition, the PWAS transducers are susceptible to damage after exposure to harsh environments. A scanning electron microscopy (SEM) was also done to visualize the microstructure of the PWAS. Figure 2 shows the cross-section of PZT-PWAS: (a) at room temperature; (b) after exposure to 250 °C temperature; and (c) after exposure to 225 kGy radiation. There are no significant changes in the microstructure or PZT grain after exposure to high temperature and radiation. Hence, PWAS can be integrated into the SHM system for successful damage detection.

## 3. Applications of PWAS for Detecting Damage in Isotropic Plates

In this section, damage detections on aluminum plates using a circular PWAS and a long PWAS were performed.

### 3.1. Corrosion Damage Detection in an Aluminum Plate Using a Circular PWAS

**Figure 3:**
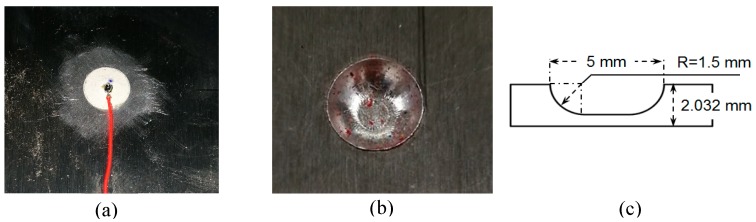
Experimental setup: (**a**) a circular PWAS; (**b**) corrosion damage; (**c**) schematic of the corrosion damage.

In this experiment, a 2.032 mm 2024-T3 aluminum plate was examined. The material properties are given in Table 1. A simulated corrosion damage was made on the 2024-T3 aluminum plate to study the damage detection by using the PWAS active sensing method, as shown in Figure 3.

**Figure 4:**
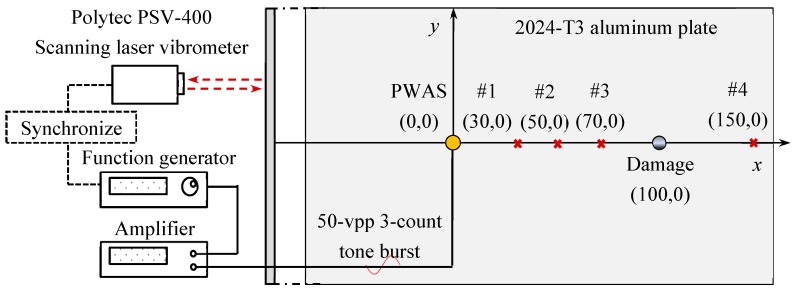
Schematic of an experimental setup using PWAS and scanning laser Doppler vibrometer (SLDV) for damage detection.

To excite Lamb waves in the aluminum plate, a circular PWAS (APC 850, 7 mm in diameter and 0.2 mm thick) was bonded on the top surface as the excitation source. Figure 4 shows the schematic of the experimental setup. The function generator was used to generate a three-count Hanning window modulated tone burst with the center frequency of 200 kHz, which was amplified to 50 Vpp by the power amplifier and applied to the circular PWAS. 

Under electrical excitation, the PWAS generates Lamb waves in the aluminum plate. Lamb waves propagate with an out-spreading pattern, interact with damage, undergo scattered and mode conversion, and are finally picked up by a Polytec PSV-400-M2 scanning laser Doppler vibrometer (SLDV). The quantity measured by the SLDV is the out-of-plane velocity of the bottom surface. Reflective tape was used to improve the signal quality. In the experiment, a line scan and an area scan on the specimen surface were carried out to detect the corrosion damage. The locations of the circular PWAS, corrosion damage, and special recording points are illustrated in Figure 4. 

#### 3.1.1. Damage Detection by using a SLDV Line Scan

**Figure 5:**
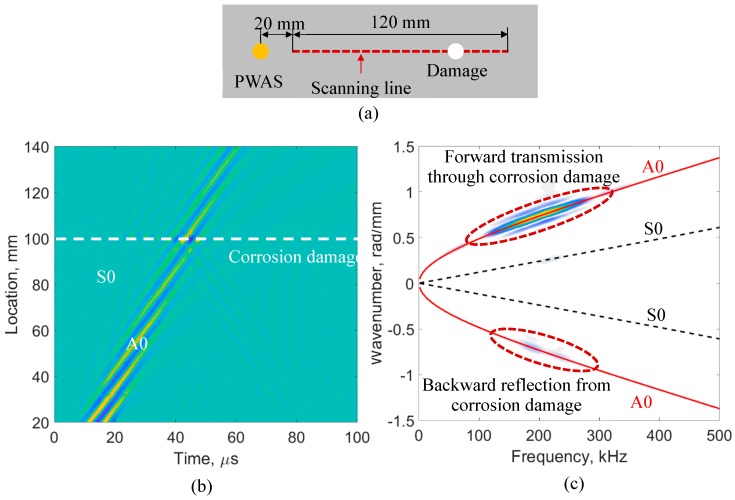
SLDV line scan for a 2.032-mm 2024-T3 aluminum plate: (**a**) schematic of the SLDV line scan; (**b**) time–space wavefield at 200 kHz; (**c**) frequency–wavenumber dispersion curves.

In the experiment, the time-space Lamb wave data was obtained from the SLDV line scan, as shown in Figure 5a. To obtain the experimental frequency–wavenumber dispersion curves, the measured time–space wavefield u(t,x) was transformed into the frequency–wavenumber domain by applying a two-dimensional (2D) fast Fourier transform (FFT). Figure 5b,c show the time–space wavefield and the corresponding frequency–wavenumber dispersion curves, the wave transmission and reflection due to the corrosion damage can be clearly observed. Hence, the damage can be easily detected by using the wavefield analysis. 

#### 3.1.2. Damage Detection by Using an SLDV Area Scan

**Figure 6:**
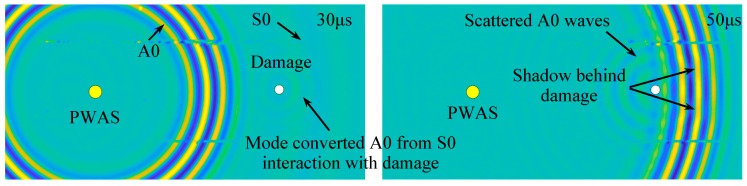
Spatial wavefield in the aluminum plate with simulated corrosion damage, showing S0 mode and A0 mode waves interacting with the corrosion damage.

In this section, an SLDV area scan was conducted to measure the wave interaction with the corrosion damage under PWAS excitation. Figure 6 shows a transient spatial wavefield in the plate. At 30 μs, the fast propagating S0 mode with a long wavelength, and the slowly propagating A0 mode with a short wavelength could be identified. The mode-converted A0 waves could be noticed, propagating with a short wavelength from S0 interaction with the damage. At 50 μs, after A0 waves interacted with damage, the scattered A0 waves could be observed, as well as the shadow behind the damage. Therefore, the damage could be visualized from the measured wavefield. 

**Figure 7:**
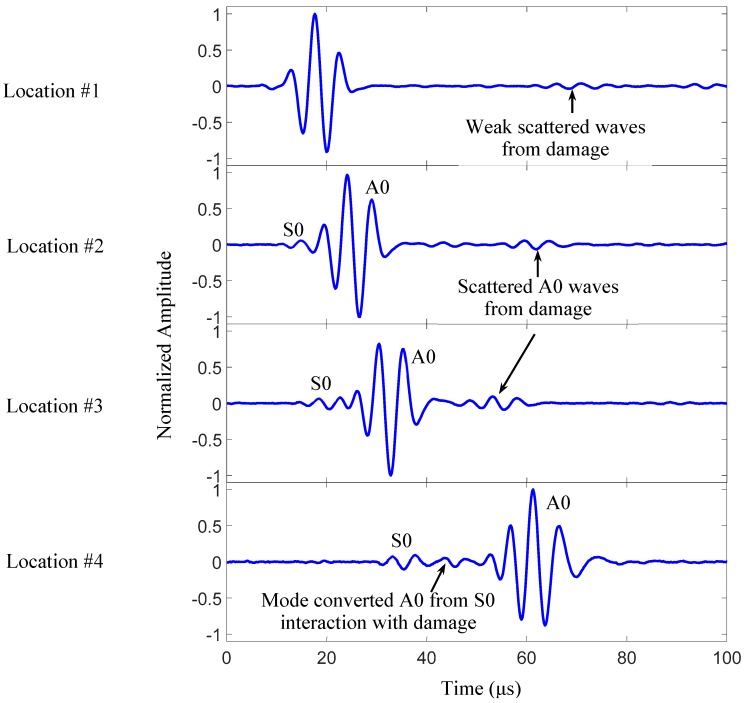
200 kHz signals of the damaged plate at locations #1 through #4, shown in Figure 4.

Figure 7 shows the waveforms at various sensing locations for the 200 kHz excitation. The signals at location #1, #2, and #3 show that the scattered A0 wave amplitude increases when the sensing location moves closer to the damage. The signal at location #4 shows the mode-converted A0 wave packet from the S0 interaction with the damage. 

### 3.2. Crack Detection in a Stiffened Aluminum Plate Using Long PWAS

This section describes an experimental procedure to analyze the scattered Lamb wave to detect a horizontal crack at the root of the stiffener in an aluminum plate.

#### 3.2.1. Experimental Procedure

**Figure 8:**
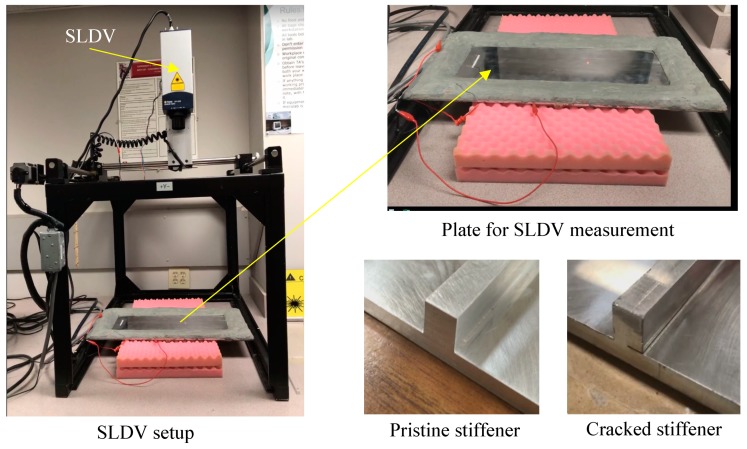
Experimental setup for the SLDV to measure the out-of-plane velocity of the scattered wavefields.

Two aluminum plates with a pristine stiffener and cracked stiffener were manufactured for the experimental study. Electrical discharge machining (EDM) method was used to create a crack along the entire length of the stiffener. The height and width of the stiffener is 8.47 mm. The thickness of the plate is 4.23 mm. For the crack stiffener, the crack width is half of the stiffener width, and the crack is present along the entire length of the stiffener (Figure 8). The experimental setup and the plates with pristine stiffener and cracked stiffener are shown in Figure 8. Two 60 mm × 5 mm × 0.2 mm PWAS transducers were bonded in a straight line on the top and bottom surfaces of the plate to create a line source. On both plates, the PWAS transducers were bonded 200 mm away from the stiffener. Two PWAS were excited simultaneously in opposite phase to generate A0 Lamb wave, selectively. The excitation signal is a three-count tone burst at 150 kHz generated by a Tektronix AFG3052C dual channel function generator. A power amplifier was used to amplify the excitation signal, which strengthens the reflected and transmitted signal from the discontinuity or damage. To create non-reflecting boundary condition, absorbing clay was applied all around the plate boundaries. 

#### 3.2.2. Experimental Results

**Figure 9:**
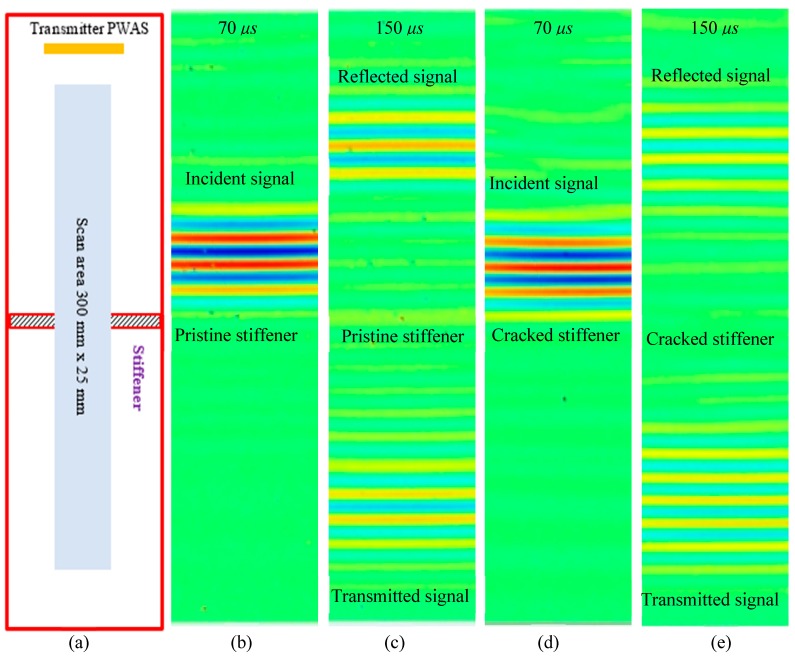
Experimentally measured scattered wavefields using SLDV: (**a**) scan schematic; (**b**) incident wave and (**c**) reflected and transmitted waves from pristine stiffener; (**d**) incident wave, and (**e**) reflected and transmitted waves from the cracked stiffener.

The wavefields were measured using SLDV. Figure 9a is the schematic of the SLDV measurement. Figure 9b–e show the reflected and transmitted wavefields for the pristine stiffener and cracked stiffener, respectively. It can be found that the long PWAS transducers can successfully generate the straight crested A0 mode Lamb wave. It also shows a minimal reflection from the plate edges, due to the use of absorbing clay. In addition, these figures show that the scattered wavefields are also straight crested waves after interacting with the discontinuity, as expected. 

**Figure 10:**
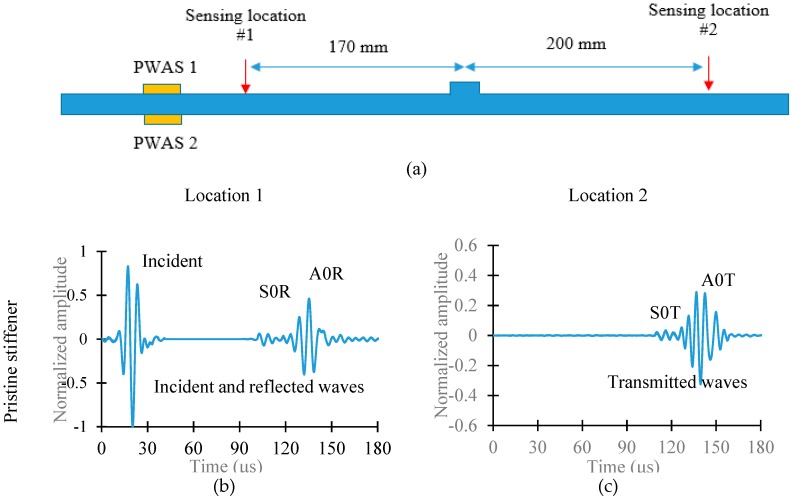

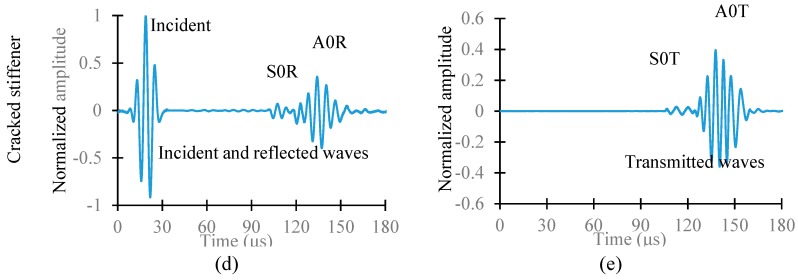
(**a**) Schematic diagram of the sensing locations; (**b**) incident and reflected Lamb waves from pristine stiffener; (**c**) transmitted Lamb waves from the pristine stiffener; (**d**) incident and reflected Lamb waves from cracked stiffener; (**e**) transmitted Lamb waves from cracked stiffener.

**Figure 11:**
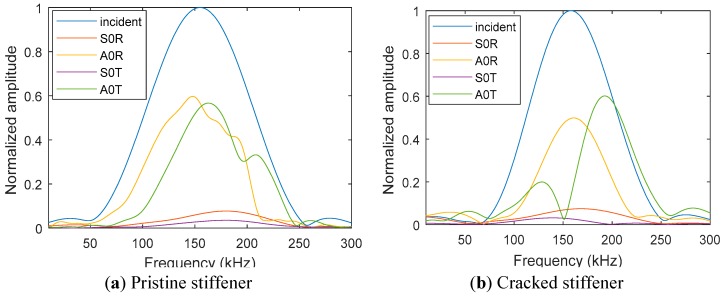
FFT of the incident and scattered Lamb waves: (**a**) pristine stiffener; (**b**) cracked stiffener.

Figure 10a shows the schematic diagram of the sensing locations. Sensing location 1 is 170 mm before the stiffener and the sensing location 2 is 200 mm after the stiffener. Figure 10b–e show the experimental scattered waveforms for the pristine and cracked stiffener, respectively. The corresponding fast Fourier transform (FFT) of the incident and scattered waves for the pristine and cracked stiffener are given in Figure 11.

The reflected and transmitted Lamb waves show a similar pattern in the time–domain signals for the pristine and cracked stiffener. However, the frequency response of the scattered signals shows a clear change in the amplitude of the scattered wavefields, due to the presence of the crack. Also, the shifting of the frequency spectrum due to the presence of the crack is an important phenomenon to note. The transmitted A0 Lamb wave has clear anti-resonance at 150 kHz for the cracked stiffener. Such information may be useful for crack detection in complex geometry.

## 4. Applications of PWAS for Detecting Damage in Anisotropic Plates

**Figure 12:**
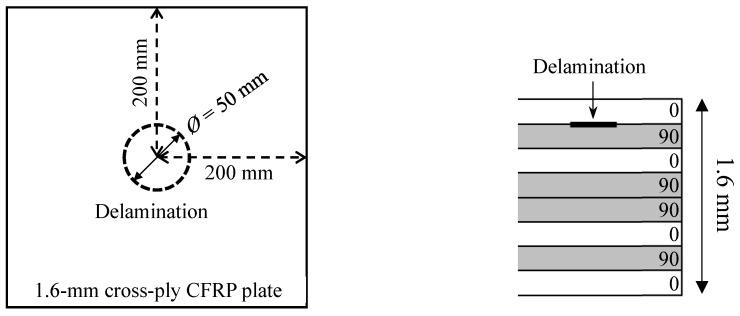
Schematic of a 1.6 mm cross-ply carbon fiber-reinforced polymer (CFRP) composite plate with circular delamination by inserting Teflon.

In this section, a 1.6 mm thick in-house cross-ply carbon fiber-reinforced polymer (CFRP) composite plate with a stacking sequence of [0/90]_2s_ was examined. The delamination is generated by inserting a 50 mm diameter Teflon film between the first ply and second ply during the ply lay-up process. Figure 12 shows the configuration of the composite plate. The engineering elastic properties of the unidirectional prepreg are given in Table 2.

A network of PWAS transducers and the damage imaging method were used to detect and quantify the delamination in the cross-ply composite plate. In recent work [70,71], an improved imaging method was developed to obtain accurate results of localization and sizing damages in metallic plates and composite laminates. Here, some more recent results in this direction are presented. The gist of our methodology [70,71] is to perform a point-by-point detection and localization of damage as a first step before using the imaging methods. Four sensors are distributed to make a cross sign on the area of interest. From the scattered waves of pulse-echo experiments, the difference in the time-of-flight (TOF) values of scattered waves of sensors are determined. If these difference values are close to zero, the damage is at the center of the area of interest. If there is difference in TOF of scattered waves, the damage location is close to the sensor that has less TOF. Based on the location of damage and sensors, the directions of the incident and scattered waves are determined. Then, the direction-dependent group velocities of incident and scattered waves for all the individual sensing paths on the composite plate are determined using the semi-analytical finite element (SAFE) approach [72]. 

### 4.1. Experimental Setup

**Figure 13:**
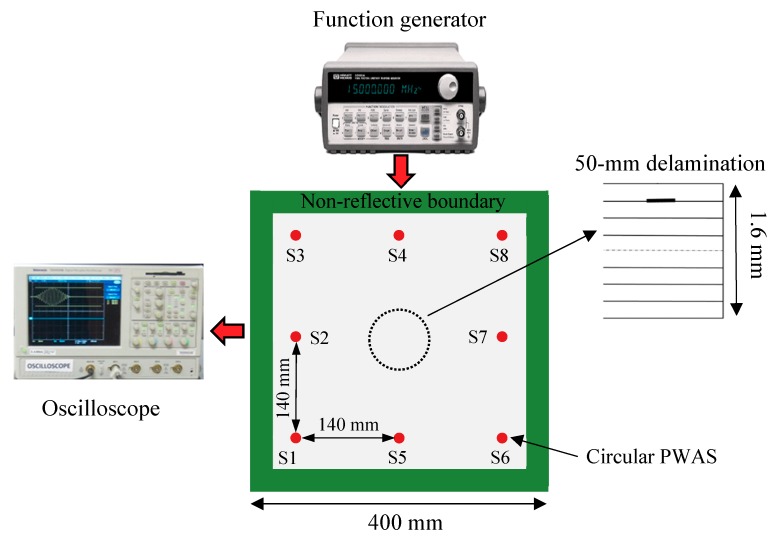
Experimental setup on the 1.6 mm cross-ply composite plate.

The experimental setup of using SHM techniques to detect the delamination in the composite plate is shown in Figure 13. Eight PWAS transducers were bonded onto the plate surface to form a sensor network. The diameter of each PWAS transducer is 7 mm, and the thickness is 0.2 mm. The clay was applied to the plate edges to absorb boundary reflections. An Agilent 33120A function generator was used to generate the excitation signal. The response signals were recorded by an oscilloscope. 

**Figure 14:**
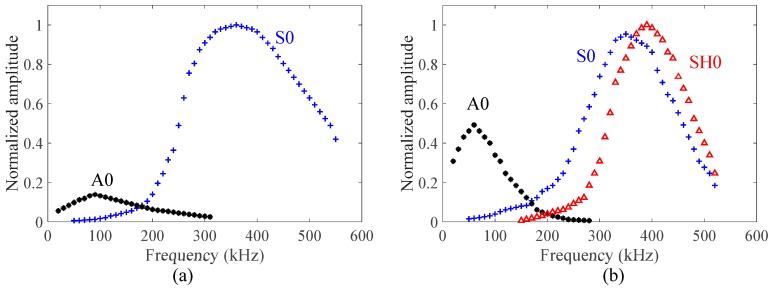
Experimental tuning curves of the cross-ply CFRP composite plate in the: (**a**) 0° directions; (**b**) 45° direction.

The excitation signal was a three-count tone burst signal at a center frequency of 330 kHz. This frequency was chosen based on the tuning curve. Experimental tuning curves of the cross-ply CFRP composite plate in the 0° and 45° directions are shown in Figure 14. It can be observed that only a single mode (S0 mode) is dominant around the 330 kHz frequency in the 0° direction. However, SH0 mode was also observed as strong as S0 mode in the 45° direction. The excitation of the SH0 wave in the off-axial direction is due to the anisotropic behavior of the composite plate already reported by Giurgiutiu [73]. 

### 4.2. Experimental Results

**Figure 15:**
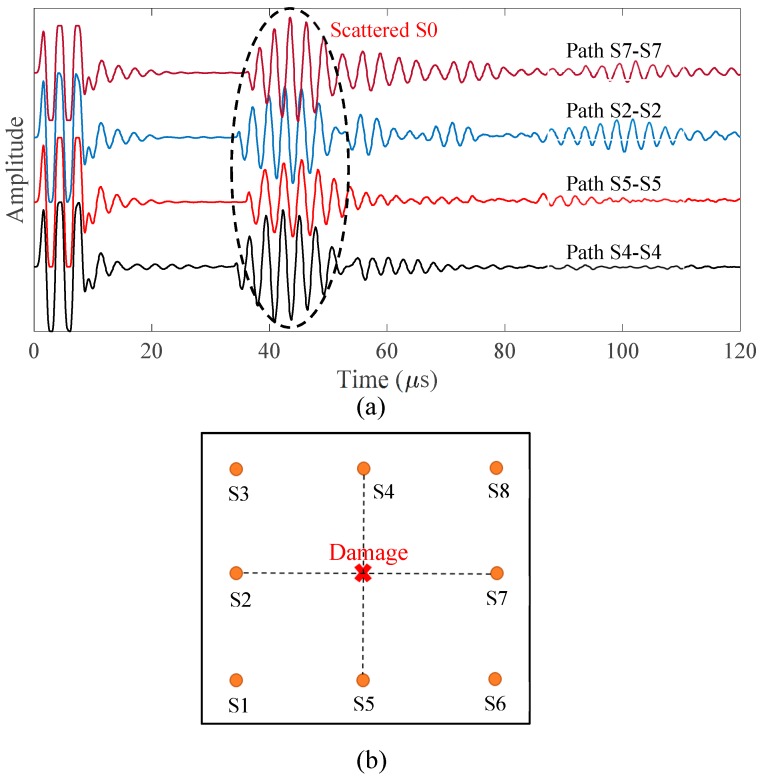
The new methodology for the localization of damage point: (**a**) pulse-echo signals of the sensing paths S2-S2, S4-S4, S5-S5, and S7-S7; (**b**) determining the location of damage.

The signals were collected by using pulse-echo and pitch-catch modes. First, the pulse-echo experiments were conducted for the PWAS S2, S4, S5, and S7 to detect and localize the damage using the proposed method in Section 4. Figure 15a shows the pulse-echo signals of the sensing paths S2-S2, S4-S4, S5-S5, and S7-S7. It can be noted that all of the signals have strong scattered S0 waves with the same TOF values. Hence, the damage is located at the center, as shown in Figure 15b. 

**Figure 16:**
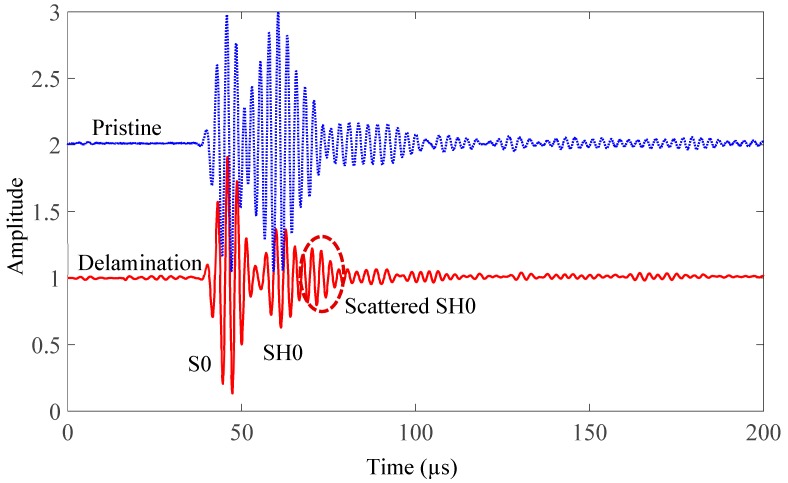
Comparison between the pristine signal and the delamination signal (Scattered S0 is overlapped with incident waves).

**Figure 17:**
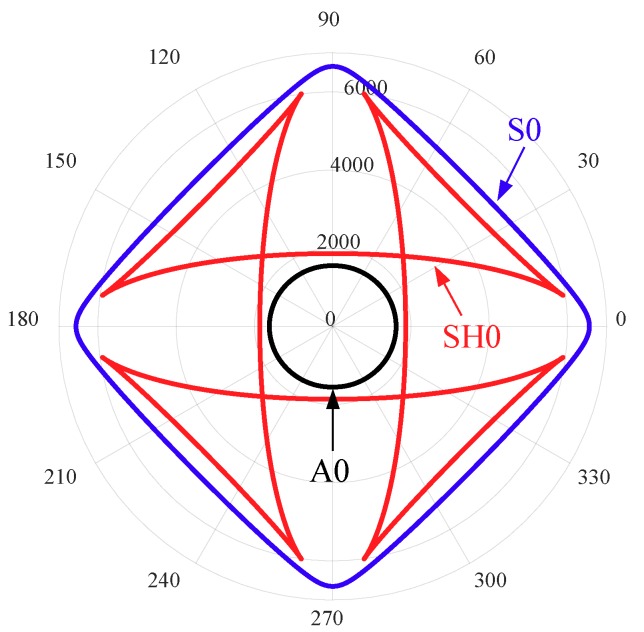
Group velocity directivity curves at 330 kHz in a 1.6 mm cross-ply CFRP plate.

In the pitch-catch mode, each PWAS transducer acted as a transmitter, whereas the rest of them in the network acted as receivers. For demonstration, only one set of signals is shown in Figure 16. In this set, PWAS S2 is the transmitter and PWAS S4 is the receiver. It can be observed that both S0 and SH0 are dominant in this sensing path (45° direction), which agrees with the experimental tuning curves in the 45° direction (Figure 14). The incident waves were determined as the S0 and SH0 modes, based on the group-velocity dispersion curve. Figure 17 shows the group-velocity directivity plot at 330 kHz. The curves indicate that Lamb waves propagate with various velocities in different directions. It can be observed that S0 mode has the highest group velocity, while A0 mode has the lowest group velocity. SH0 mode possesses a group velocity between A0 and S0, and it shows self-crossing behavior, as reported in Glushkov [74]. The scattered SH0 wave, due to the delamination, can be determined by calculating the TOF of incident path (PWAS S2 to damage) and damage path (damage to PWAS S4) using the corresponding group velocities and distances.

Based on the time of flight (TOF) of scattered SH0 waves, the imaging method can be used to detect and quantify the delamination in the plate. The basic idea of the imaging method is to divide the interested area into pixels, and to find the field values of these pixels for each sensing path. The TOF of every pixel can be determined by using Equation (1):

(1) $$t_{ij}=\frac{\sqrt{{\left(x_{T}-x_{i}\right)}^{2}+{\left(y_{T}-y_{j}\right)}^{2}}}{v_{g1}}+\frac{\sqrt{{\left(x_{i}-x_{R}\right)}^{2}+{\left(y_{j}-y_{R}\right)}^{2}}}{v_{g2}}$$

where $t_{ij}$ is the TOF of every pixel, and $x_{T},\;y_{T},\;x_{R},\;y_{R},\;x_{i},\;y_{j}$ are the coordinates of the transmitter PWAS, receiver PWAS, and pixel, respectively. $v_{g1}$ and $v_{g2}$ are the group velocities of the incident path (transmitter PWAS to damage) and damage path (damage to receiver PWAS), respectively. These group velocities are determined from Figure 17, based on the direction of the incident and scattered waves. When the pixels lie on the damage orbit of a particular sensing path, which means $t_{ij}=t_{d}$ ($t_{d}$ is the TOF of scattered wave). In this case, the field value of these pixels is maximum.

**Figure 18:**
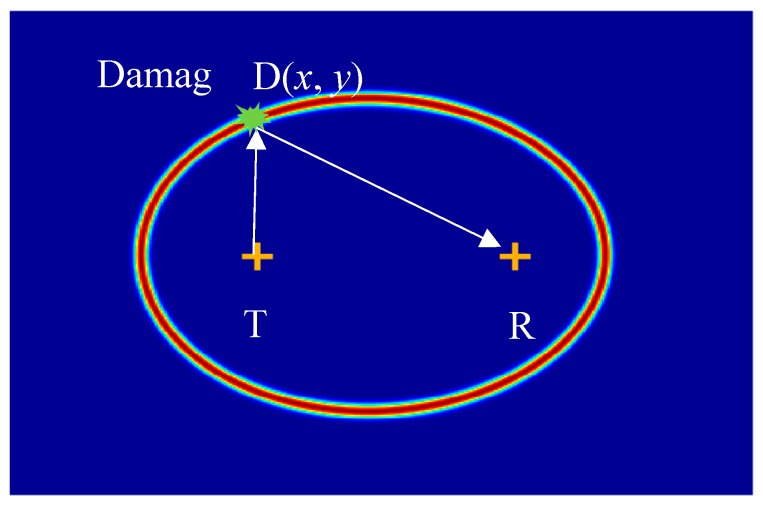
Damage orbit with the transmitter and receiver PWAS as foci.

**Figure 19:**
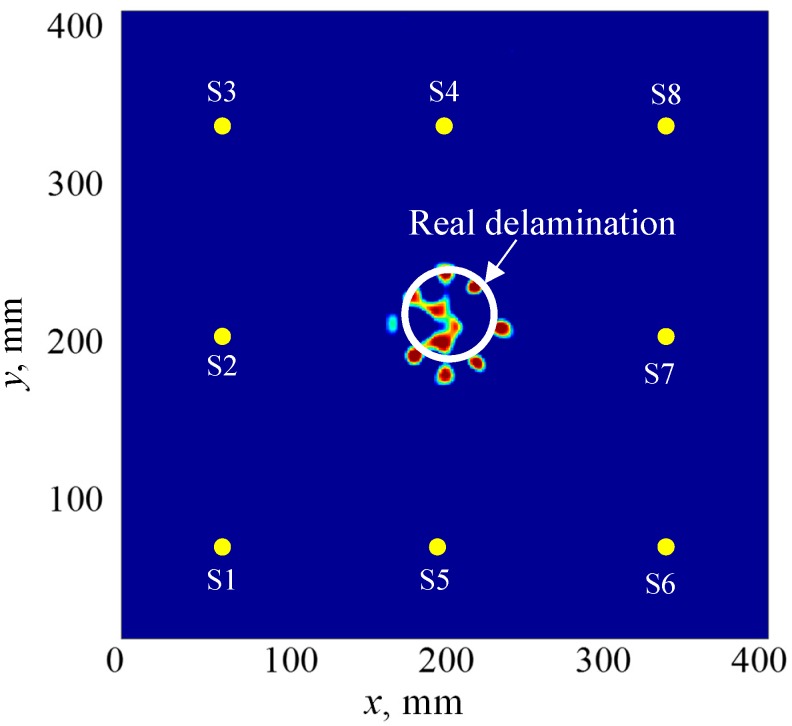
The imaging result of the 50 mm delamination in the cross-ply composite plate.

Figure 18 gives the damage orbit for a certain sensing path. To determine the size of the delamination area, the sensing paths of multiple transmitters around the area of interest were used to get multiple intersection points that represent the damage edges. A new methodology was implemented to visualize the delamination without setting a threshold, using a combination of summation and multiplication algorithms. In this methodology, the summation algorithm [71] is used to extract the individual image of all the sensing paths for each PWAS transmitter. These images have strong intersection points, which represent the delamination edges, and the rest are undesirable orbits. The multiplication algorithm is used to fuse all of these individual images, to obtain the final image for the delamination, which does not require setting of a threshold. Figure 19 shows the final imaging result of the delamination. It can be found that the results of the imaging method match well with the real delamination. The edges of the 50 mm delamination can be observed. 

## 5. PWAS as an AE Sensor for In Situ AE Detection during a Fatigue Crack Event

Application of PWAS as an acoustic emission (AE) sensor is presented in this section. AE events in metallic or composite materials can be generated, due to various phenomena such as friction, plastic deformation, crack growth event, etc. Of all the causes, fatigue crack growth is a problem of great importance in metals. 

**Figure 20:**
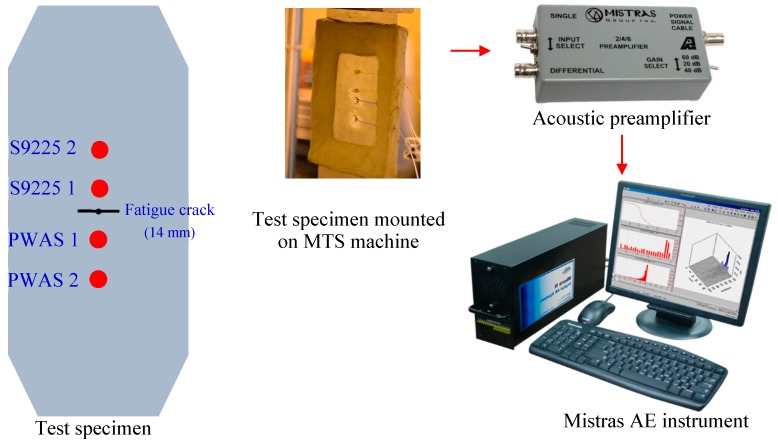
Experimental setup for capturing AE signals during the fatigue crack event.

To understand the sensing capability of PWAS to detect AE signals due to a crack growth event, PWAS transducers were used as an in situ sensor on a high-strength aluminum 2024-T3 specimen while undergoing fatigue crack growth. The aluminum specimen was 103 mm in width, 305 mm in, and 1 mm thick. A 1 mm hole was drilled at the geometric center of the specimen. Fatigue loading was applied on the specimen to generate a pre-crack of 14 mm tip-to-tip length. For the pre-crack generation, fatigue loading from 13.85 kN to 1.38 kN at a frequency of 10 Hz was applied. After initiating the crack approximately up to 14 mm tip to tip, two PWAS transducers at 6 mm and 25 mm from the hole, as well as two S9225 sensors at 6 mm and 25 mm from the hole were installed on the aluminum plate, as shown in Figure 20. S9225 is a commonly used and commercially available AE sensor. The S9225 sensor was used to make a comparison between PWAS and S9225 in this experiment. The test specimen installed with PWAS and S9225 transducers was mounted on the MTS machine. The fatigue loading was continued to vary between 13.85 kN and 1.38 kN with a loading rate of 2 Hz, and simultaneous AE measurements were performed. The experimental setup for capturing the AE signal from the fatigue crack growth is presented in Figure 20. AE signals during the crack growth were captured by using PWAS and S9225 sensors. The sensors are connected to the acoustic preamplifier. The acoustic preamplifier is a bandpass filter, which can filter out signals between 30 kHz to 700 kHz. The preamplifier is then connected to a four-channel Mistras AE system for processing the signals.

**Figure 21:**
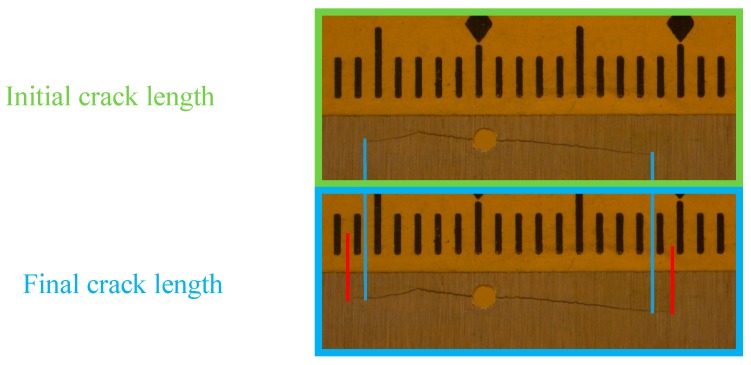
The initial tip-to-tip crack length was approximately 14 mm. After 2000 fatigue cycles, an advancement in the crack length of 2 mm was observed.

The crack growth on the specimen due to 2000 fatigue loading cycles from 14 mm to 16 mm is presented in Figure 21. The initial crack is presented in the green box, and the final crack in the blue box. The locations of the initial crack tips were marked using the blue lines, and the locations of the final crack tips were marked using red lines. The crack grew by approximately 2 mm. AE signals were captured simultaneously during the fatigue crack growth. 

**Figure 22:**
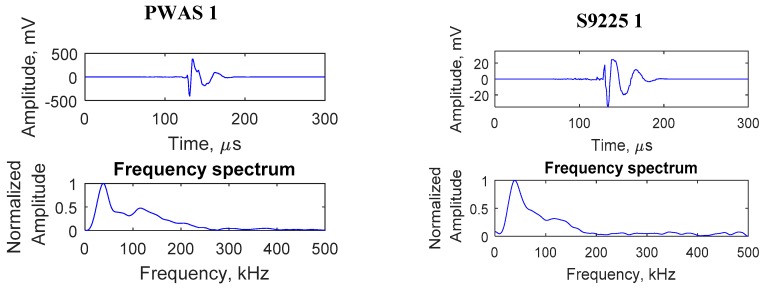
Signals captured by the PWAS 1 and S9225 1 sensors, due to a particular AE event at the crack.

A particular AE event captured by PWAS 1 and S9225 1 sensors is presented in Figure 22. The relative time of arrival of the signals at the PWAS 1 and S9225 1 sensors installed equidistant from the crack was obtained from the Mistras AE system. The time of arrival of signals at PWAS 1 and S9225 1 was found to be the same, which confirmed that the signals corresponded to an AE event happening that was equidistant from the sensors, which is the crack location. If the AE event was happening at a different location other than the crack, the time of arrival at the PWAS 1 and S9225 1 sensors would be different. In this way, it was confirmed that the AE signal captured was originating from the crack.

**Figure 23:**
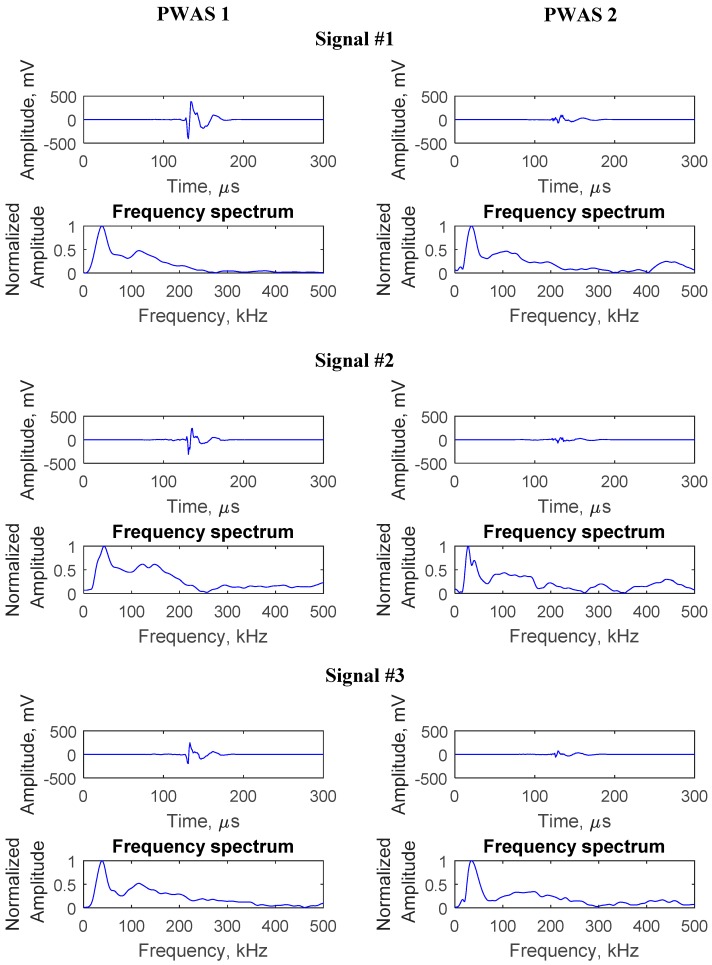
Typical AE signals captured during fatigue crack growth. Three sets of captured signals are presented. For each set, the signal at PWAS 1 and the corresponding signal at PWAS 2 are presented. The AE signals seem to maintain their signature at PWAS 1 and 2.

Several AE signals originating from the crack were captured during this crack growth event, as shown in Figure 23. From the time of arrival of the AE signals at PWAS 1 and PWAS 2, signals corresponding to the same AE event were identified. The sequence of arrival, as well as the amplitude of the AE signals, are presented in Table 3. The sequence of arrival confirms that the AE signals were originating from the crack. Geometric spreading caused the amplitude of AE signal to decay while propagating. This was also observed from the corresponding amplitude values of the signals at PWAS 1 and PWAS 2. The AE signals were found to have a similar signature by comparing the waveform, as well as the frequency spectrum of the signals. It can be observed that the frequency spectrum maintains its signature when reaching PWAS 2.

From the experimental investigation presented in this section, it can be concluded that PWAS can be used as an in situ sensor for detecting AE signals during the fatigue crack growth. Fatigue loading was applied on the specimen, to grow the crack, and simultaneous measurement of AE signals was performed. The time of arrival of AE signals at multiple sensors confirms that the AE signals are originating from the crack. The amplitude decay of AE signals due to geometric spreading was also observed through AE measurements at PWAS transducers located at 6 mm and 25 mm from the crack.

## 6. Conclusions

In this paper, PWAS endurance in harsh temperatures and radiation environments, and PWAS active and passive sensing methods for SHM applications were demonstrated. First, the characterization of PWAS was conducted. No significant change in the microstructure or PZT grain after exposure to high temperature and radiation was found through the SEM technique. Proper characterization of PWAS allows a SHM system to infer the integrity of the transducers and separate transducer-flawed signals from structural defects in a harsh environment. For PWAS active sensing methods, PWAS was successfully used to detect the simulated crack and corrosion damage in aluminum plates, and simulated delamination damage in the composite plates. The frequency response of the scattered signals showed a clear change in the amplitude, due to the presence of the crack. The improved imaging method does not require a threshold to obtain the final image for the damage visualization. For the PWAS passive sensing methods, PWAS was successfully used as AE sensors for in situ AE detection during fatigue crack growth. The time of arrival of AE signals at multiple sensors confirms that the AE signals were originating from the crack. The amplitude decay of the AE signals due to geometric spreading was validated.

## Figures and Tables

**Table 1:** Material properties of 2024-T3 aluminum.

Young’s Modulus (*E*)	Poisson’s Ratio (*υ*)	Density (*ρ*)
73.1 GPa	0.33	2780 kg/m^3^

**Table 2:** Engineering constants of the unidirectional prepreg.

*E*_11_	*E*_22_	*E*_33_	*ν*_12_	*ν*_13_	*ν*_23_	*G*_12_	*G*_13_	*G*_23_	*ρ*
140.8 GPa	11.3 GPa	11.3 GPa	0.31	0.31	0.5	5.7 GPa	5.7 GPa	3.4 GPa	1640 kg/m^3^

**Table 3:** Sequence of arrival and amplitude of the AE signals at PWAS 1 and PWAS 2.

Signal #	Sequence of Arrival		Amplitude of Signal (dB)	
	PWAS 1	PWAS 2	PWAS 1	PWAS 2
1	1	2	62	51
2	1	2	59	49
3	1	2	58	48
